# A Decade of Lung Transplantation in China (2015–2024): Driving Quality Improvement and Scale Expansion Through Management Systems

**DOI:** 10.3390/healthcare14142075

**Published:** 2026-07-10

**Authors:** Xiao-Shan Li, Chun-Xiao Hu, Gong-Tao Qian, Wei-Wei Xu, Yi Lu, Jing-Yu Chen

**Affiliations:** 1 Department of Lung Transplantation Center, The Affiliated Wuxi People’s Hospital of Nanjing Medical University, Wuxi People’s Hospital, Wuxi Medical Center, Nanjing Medical University, Wuxi 214023, China; sclxs87@163.com (X.-S.L.); huchunxiao91211@163.com (C.-X.H.); qiangongtao@sina.com (G.-T.Q.); xyfylhy@163.com (W.-W.X.); akashi0424@163.com (Y.L.); 2 China Quality Management and Control Center for Lung Transplantation, Wuxi 214023, China

**Keywords:** lung transplantation, organ donation, healthcare management, quality control, standardization, China experience

## Abstract

Since organ donation after citizen death became the sole legal source in 2015, lung transplantation (LTx) in China has undergone a systematic transformation, shifting from numerical scarcity to scale expansion and from technical exploration to sustained improvement in quality. From a management perspective, this article provides a comprehensive review of the development of LTx in China between 2015 and 2024, demonstrating that its central achievement has been the establishment of an integrated management system that combines national top-level policy design, standardized and homogeneous training, multidisciplinary collaboration, standardized clinical pathways, and rigorous quality assessment. Supported by a mechanism based on data-driven decision making, standard-guided implementation, and closed-loop management, this system has effectively ensured quality homogeneity and controllable risk during the dissemination of advanced transplantation techniques. Over the past decade, the number of hospitals performing LTx increased from 9 to 45, annual procedure volume rose from 118 to 925 cases, and the cumulative total of completed LTx was 5483. During the same period, 30-day survival improved from 78.5% to 85.3%. This system-oriented management model provides an important reference for the global community, particularly for developing countries facing challenges related to resource imbalance, by demonstrating how institutional innovation can facilitate the safe and efficient dissemination of highly complex medical technologies.

## 1. Introduction

### 1.1. A Decade of Concurrent Opportunities and Challenges

Lung transplantation (LTx) is the only effective treatment for end-stage lung disease. In 2015, when organ donation after citizen death became the sole legal source of organs for transplantation in China, the national LTx program entered a new stage of development. This transition followed a series of legislative and ethical reforms, including the 2007 State Council Regulation on Human Organ Transplantation, the 2011 Amendment (VIII) to the Criminal Law that criminalized organ trafficking, and the public commitment announced in 2005 to gradually replace reliance on organs from executed prisoners with voluntary, unpaid citizen donation [1,2]. Since 2015, all donated organs have been required to originate from voluntary donation after citizen death, with allocation conducted through the mandatory computerized China Organ Transplant Response System (COTRS). For lung transplantation, COTRS-based allocation was implemented in October 2018 (Table 1). The historical development of organ procurement in China has been the subject of international debate, including concerns regarding data transparency, donor traceability, and independent oversight. A comprehensive analysis of these issues has been provided in recent publications addressing the evolution of the organ donation framework in China, including analyses involving international experts [1,2,3]. This review focuses on the management systems that have shaped clinical LTx practice since the implementation of the donation reform.

At the beginning of this transition, LTx in China was still in an early stage of development. Only 118 procedures were performed nationwide, and only 9 institutions were capable of providing LTx [4]. Clinical practice also faced substantial challenges, including variability in donor lung quality, a high proportion of elderly and critically ill recipients, and pronounced regional disparities in medical resources. In this highly technical and high-risk field, achieving a transition from initial scale expansion to quality improvement, and from isolated center-based exploration to systematic nationwide implementation, emerged as a central challenge in healthcare management.

By contrast, LTx in North America and Europe had evolved over several decades within established regulatory and registry frameworks, including the Organ Procurement and Transplantation Network (OPTN), the Scientific Registry of Transplant Recipients (SRTR), and the International Society for Heart and Lung Transplantation (ISHLT) Thoracic Transplant Registry. These systems demonstrated the importance of registry-based surveillance, structured organ allocation, and standardized clinical pathways in maintaining program stability and improving outcomes [5,6,7]. For China, the central issue was therefore not whether such frameworks were necessary, but how they could be adapted and implemented at a national level within a distinct legal, ethical, and healthcare environment and within a relatively short period.

**Figure 1:**
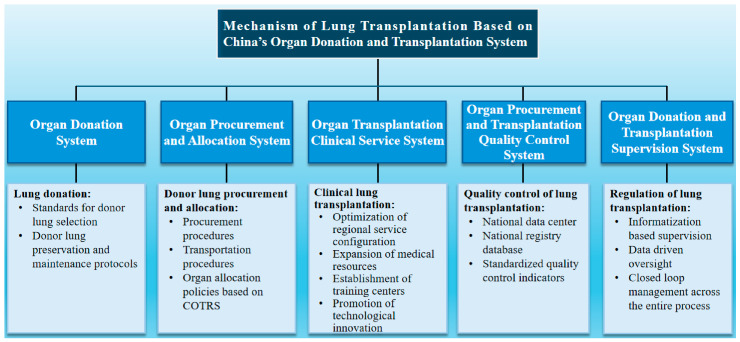
Operational Mechanism of Lung Donation and Transplantation in China. This flowchart delineates the five core, interconnected domains of the Chinese lung transplantation system: (1) Organ Donation, (2) Organ Procurement and Allocation, (3) Clinical Service, (4) Quality Control, and (5) Supervision. It illustrates the sequential and integrated pathway from donor selection through organ allocation via the China Organ Transplant Response System (COTRS), clinical transplantation, to nationwide quality monitoring and data-driven oversight, forming a closed-loop management system.

During the past decade, LTx in China has been organized around five core domains: donation, organ procurement and allocation, clinical services, quality control, and regulation [8,9]. This structure enabled the progressive establishment of a comprehensive and professionally differentiated donation and clinical care system, as shown in Figure 1. Standardized criteria for donor lung selection and management were defined [10], and standardized procedures for procurement and transportation [11,12], together with equitable allocation, were implemented through the China Organ Transplant Response System (COTRS) [13,14]. Clinical capacity and accessibility were enhanced through optimization of regional service networks, expansion of resource supply, development of specialized training centers, and promotion of technological innovation. A standardized quality indicator system was established and implemented under the National Quality Management and Control Center for Lung Transplantation, hereafter referred to as the Quality Control Center, and the National Lung Transplantation Data Center, hereafter referred to as the Data Center [15]. In parallel, information-based and data-driven regulatory oversight was advanced to achieve closed-loop management from donation through transplantation, thereby protecting the legitimate rights and interests of all stakeholders [16].

Although LTx in China began relatively late, sustained policy guidance and systematic capacity building over the past decade have created unprecedented opportunities for development and have placed the field on a trajectory of coordinated improvement in both quality and scale [17].

### 1.2. Scale Expansion: Evidence from National Data

Over the past decade, lung donation and transplantation in China have increased in both volume and quality. The national figures presented here are drawn from the China Lung Transplantation Registry (CLuTR) and the annually published Report on Organ Donation and Transplantation in China, which together constitute the principal traceable sources of nationwide donation and transplantation activity [18,19]. The average lung yield per donor rose from 0.15 in 2019 to 0.26 in 2024. The number of institutions accredited to perform LTx increased from 26 in 2015 to 60 in 2024, and the number of hospitals actively performing LTx increased from 9 to 45. Nationwide annual transplant volume rose steadily from 118 procedures in 2015 to 925 in 2024, with a cumulative total of 5483 procedures over ten years. During the same period, 30-day survival increased from 78.5% to 85.3%. In the most recent national cohort with available follow-up data, recipient survival was 61.1% at one year, 48.9% at three years, and 41.8% at five years (Table 1). The national retransplantation rate was 2.8%, and the reported rates of infection, acute rejection, and airway anastomotic complications were 66.1%, 6.9%, and 8.8%, respectively. Donor characteristics provide additional context for interpreting these outcomes. The median donor age was 42 years (interquartile range, 32.0 to 49.0), and most donors were male (84.6%). The leading causes of donor death were cerebrovascular disease (51.9%), traumatic brain injury (40.0%), and others (8.1%). The median duration of ventilator support before procurement was five days, and 3.2% of donors had a documented history of aspiration. The increasing use of extracorporeal membrane oxygenation (ECMO) reflects the growing complexity of recipient management. Seven percent of recipients were bridged to transplantation with ECMO, and intraoperative ECMO support was used in 67.4% of procedures. Thirty-day survival was 89.3% among recipients who did not require intraoperative ECMO and 81.2% among those who did (*p* < 0.001), consistent with the greater clinical complexity among patients requiring mechanical circulatory or respiratory support. These findings reflect the expansion of LTx to a broader population of patients with advanced lung disease and the development of a more comprehensive national transplant service.

**Table 1:** Donation- and Transplantation-Related Indicators of Lung Transplantation in China, 2015 to 2024.

Indicator		2015	2016	2017	2018	2019	2020	2021	2022	2023	2024
Number of patients on the lung transplant waiting list at year end ^†^ [18]		-	-	-	-	89	147	205	273	352	262
Mean number of lungs procured per donor ^†^ [18]		-	-	-	-	0.15	0.18	0.27	0.26	0.28	0.26
Number of accredited transplant hospitals [20]		26	26	29	31	43	43	49	54	60	60
Number of hospitals performing lung transplantation [18]		9	12	14	15	23	29	32	36	43	45
Number of lung transplant procedures [18]		118	204	299	403	489	513	775	798	959	925
Retransplantation rate/% [19]		2.8									
Infection rate/% [19]		66.1									
Acute rejection rate/% [19]		6.9									
Airway anastomotic complication rate/% [19]		8.8									
Overall survival rate/% [19]	30-day	78.5				84.63	83.5	83.7	83.4	84.7	85.3
	1-year	62.4					60.6				
	3-year	50.1					47.8				
	5-year	41.7					43.1				
Five high-volume centers survival rate/% [19]	30-day	84.2									
	1-year	62.9									
	3-year	50.1									
	5-year	43.0									
Other (lower-volume) centers survival rate/% [19]	30-day	82.8									
	1-year	56.7									
	3-year	44.7									
	5-year	39.4									

^†^ Note: The China Organ Transplant Response System for lung allocation was implemented in October 2018. Retransplantation, infection, acute rejection, and airway anastomotic complication rates are reported as national aggregate values from the China Lung Transplantation Registry (CLuTR). Survival figures by stratum reflect the most recent reportable cohort; center-specific outcomes are not disclosed individually, and survival stratified by center volume is presented instead to illustrate cross-center consistency. Chronic lung allograft dysfunction (CLAD)-free survival is not yet captured as a discrete field in the national registry and is therefore not reported.

In an international context, the pace of this expansion has been notable. In the United States, adult LTx volume reached 3049 procedures in 2023 within a relatively stable network of transplant centers. Over the same period, waiting-list mortality declined to 13.3 deaths per 100 follow-up years, and 1-year post-transplant survival remained approximately 88.5% for the 2022 transplant cohort [5,6]. Data from the ISHLT registry show a similar global trend, with annual LTx activity increasing from the early 1990s to approximately 3800 procedures in 2019 and 1-year survival improving from 75.8% in the 1992–2000 cohort to 88.4% in the 2018–2023 cohort [7].

The COVID-19 pandemic disrupted transplant activity worldwide, with global LTx volume declining by approximately 11% in 2020 [7]. In contrast, transplant activity in China continued to increase modestly, from 489 procedures in 2019 to 513 in 2020. This pattern suggests that the evolving national management framework may have contributed to operational continuity during a period of substantial healthcare disruption. Against this background, the increase in China from 118 procedures in 9 active centers in 2015 to 925 procedures in 45 centers in 2024 represents a rapid national expansion in LTx capacity. Improvements in perioperative outcomes accompanied this expansion, with 30-day survival increasing from 78.5% in 2018 to 85.3% in 2024.

International comparisons should be interpreted cautiously. One-year survival reported from China (61.1%) remains lower than the approximately 88% reported in recent ISHLT and OPTN/SRTR cohorts [5,6,7], with a greater difference observed at three and five years. Several factors may contribute to this difference, including the high proportion of critically ill recipients requiring ventilatory or ECMO support, the relative limitations of long-term follow-up infrastructure, and differences in recipient and donor characteristics. In addition, several internationally used indicators, including chronic lung allograft dysfunction (CLAD)-free survival, are not yet available from the national registry. Nevertheless, a longitudinal perspective reveals that China has achieved concurrent growth in transplant volume and perioperative survival, indicating that clinical quality has not been compromised by program expansion. These advances underscore the pivotal role of coordinated management frameworks in supporting the development of LLTx in China, and they also provide a foundation for further progress toward international benchmarks.

Understanding how China expanded LTx capacity while maintaining clinical quality requires examination of the management framework underlying this transformation. Five interrelated components form the foundation of the current system: (1) national quality control and data infrastructure, (2) standardized training systems, (3) multidisciplinary team requirements, (4) technical standards and clinical pathways, and (5) quality evaluation and dynamic exit mechanisms. Together, these components support coordinated governance across the transplantation process, from donor evaluation to long-term recipient follow-up. The following sections discuss each component in detail, and Supplementary Table S1 summarizes key policy milestones, institutional responsibilities, accreditation criteria, and technical standards.

## 2. Building a Management System: Five Pillars of the Chinese Experience

**Figure 2:**
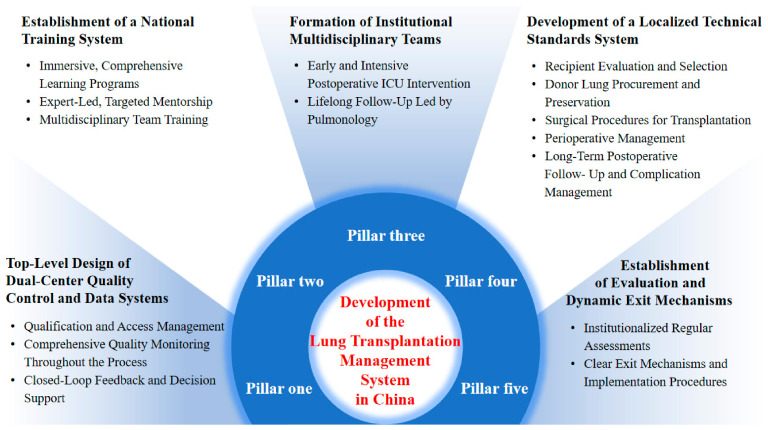
The Five Pillars of the Lung Transplantation Management System in China. This schematic diagram summarizes the core components of the management framework: (1) National Quality Control and Data Centers, (2) Homogeneous Training System, (3) Institutionalized Multidisciplinary Teams, (4) Standardized Technical Pathways, and (5) Dynamic Evaluation and Exit Mechanisms. These pillars interact to support the safe scale-up of lung transplantation.

The sustained expansion of LTx in China has not occurred in isolation. Rather, it has been supported by a systematically designed and progressively implemented management framework consisting of five core pillars, as summarized below and shown in Figure 2.

### 2.1. Pillar One: Top-Level Design of National Quality Control and Data Centers

National-level systematic planning constitutes the institutional foundation for the standardized and regulated development of LTx. A central component of this top-level design was the establishment of two functionally complementary and closely coordinated entities: the Quality Control Center and the Data Center.

The Quality Control Center was established in 2016 under the leadership of the former National Health and Family Planning Commission and is responsible for nationwide supervision, evaluation, and continuous improvement of the quality of LTx services. The Data Center was initiated in 2010 under the former Ministry of Health, with core responsibilities that include standardizing data definitions, clarifying reporting procedures, and developing a national clinical database for LTx. Both centers operate through leading clinical institutions in China, such as Wuxi People’s Hospital, thereby integrating administrative oversight with professional technical support. Their management functions are reflected in three key domains (detailed in Supplementary Table S1).

First, accreditation and access management: In accordance with the *Regulations on the Clinical Application Management of Human Organ Transplantation Technology (2020 edition),* strict entry criteria are applied to medical institutions seeking to perform LTx. These criteria encompass infrastructure requirements, multidisciplinary team capacity, including surgery, respiratory and critical care medicine, anesthesia, and intensive care, as well as professional qualifications of personnel, thereby ensuring service homogeneity at the point of entry [21].

Second, comprehensive quality monitoring: Through the establishment and implementation of the China Lung Transplantation Registry (CLuTR), real-time reporting of key data for all transplant procedures is achieved [19]. The registry enables dynamic monitoring and analysis of core quality indicators, including cold ischemia time, rates of postoperative complications, and recipient survival, thereby supporting continuous quality surveillance.

Third, closed-loop feedback and decision support: On the basis of statistical analyses conducted by the Data Center, individualized quality feedback reports are provided to transplant centers to facilitate targeted quality improvement and to establish an internal closed-loop process encompassing data collection, analysis, feedback, and optimization. In parallel, national-level quality control reports are generated at regular intervals to refine and update quality indicators and to provide empirical evidence to inform policy adjustments, resource planning, and regulatory oversight by health authorities.

The establishment of this dual-center framework represents a shift in the governance of LTx in China from a fragmented, experience-based approach to a centralized, data-driven model, thereby providing a robust institutional foundation for subsequent expansion in scale and sustained improvement in quality. The framework resembles established international models such as the OPTN-SRTR partnership in the United States, in which policy development, organ allocation, and registry-based analysis are coordinated through complementary organizations [5,6]. One feature of the Chinese model is the close integration of administrative oversight with operational practice within leading transplant centers. This arrangement shortened the interval between data collection, standard revision, and clinical implementation. During a period of rapid expansion, such coordination allowed policy and technical standards to be adapted in response to evolving clinical and operational conditions rather than relying primarily on retrospective analysis.

### 2.2. Pillar Two: Establishing a National Training System to Promote Homogeneous Development of Technical Capacity

To systematically address substantial disparities in LTx expertise across medical institutions and to reduce patient risk associated with technical variability, China has progressively established and refined a standardized training and certification system for LTx physicians [22,23]. This system is anchored in training bases accredited by national health administrative authorities and currently includes four institutions: Wuxi People’s Hospital, the China–Japan Friendship Hospital, the Second Affiliated Hospital of Zhejiang University School of Medicine, and the First Affiliated Hospital of Guangzhou Medical University. Collectively, these centers have trained and deployed more than 230 LTx surgeons and over 1500 multidisciplinary professionals to transplant programs nationwide.

Sing the National Lung Transplantation Training Base at Wuxi People’s Hospital as an illustrative example, the training framework demonstrates several defining features.

First, immersive, full-process training: The training period is typically one year, during which trainees are fully integrated into the clinical workflow of the host institution. Participants engage across the entire continuum of care, including donor evaluation and maintenance, organ procurement, transplantation surgery, and perioperative and long-term management, thereby achieving comprehensive, practice-based learning.

Second, expert-led, precision mentorship: Training is delivered under the direct supervision of a leading expert team, represented by Professor Jingyu Chen, ensuring rigorous, hands-on instruction and the accurate transmission of core surgical techniques, perioperative decision-making principles, and quality control concepts.

Third, multidisciplinary, team-based training: The program extends beyond surgeons to include professionals from thoracic surgery, respiratory and critical care medicine, anesthesiology, intensive care medicine, and specialized nursing. This approach is designed to strengthen coordinated team performance and integrated clinical decision making.

This standardized training model has effectively reduced geographic and institutional barriers to the dissemination of key technical skills and management experience, enabling the efficient replication of systematic solutions from leading centers to emerging transplant programs. It represents a core mechanism through which China has achieved nationwide homogenization of LTx capacity and has provided a robust workforce foundation for sustained improvements in surgical quality and for the safe, orderly expansion of the field.

### 2.3. Pillar Three: Standardizing Coordinated Care Through Institutionalized Multidisciplinary Team Development

The success of LTx depends not only on the surgical procedure itself but also to a large extent on systematic, highly coordinated perioperative and long-term management. To address this requirement, China has implemented mandatory regulatory policies, including the *Regulations on the Clinical Application Management of Human Organ Transplantation Technology (2020 edition)*, which explicitly require transplant centers to establish structurally complete and consistently functioning multidisciplinary teams [21]. Core members of these teams routinely include specialists in thoracic surgery, respiratory and critical care medicine, anesthesiology, and intensive care medicine, with extended participation from cardiology, rehabilitation medicine, clinical nursing, nutrition, and psychology. This institutional framework elevates multidisciplinary collaboration from a recommended practice to a prerequisite for program accreditation, thereby ensuring continuity and integration across the care pathway. In practice, implementation of this pillar has focused on two key areas.

First, early and intensive engagement in postoperative critical care: Regulations require intensive care physicians to participate throughout preoperative evaluation and to assume primary responsibility for early postoperative management in the intensive care unit, including life support, maintenance of multiorgan function, and infection prevention and control. Among lung transplant recipients in China, the proportion of patients admitted to the intensive care unit before transplantation for clinical optimization increased from 9.4% in 2019 to 18.1% in 2024 [18]. This increase reflects both enhanced capacity to manage critically ill candidates and the increasingly central role of intensive care medicine in perioperative management.

Second, lifelong follow-up led by respiratory medicine: Physicians in respiratory and critical care medicine are assigned primary responsibility for long-term care, including lifelong follow-up, individualized adjustment of immunosuppressive regimens, and the diagnosis and management of late complications such as acute rejection and chronic lung allograft dysfunction. Authoritative guidance for these practices is provided by national consensus documents issued by the Branch of Organ Transplantation of the Chinese Medical Association, including the *Diagnosis and Treatment Specification for Immunosuppressive Therapy and Rejection of Lung Transplantation in China (2019 edition)* [24] and the *Technical Specification for Diagnosis and Treatment of Complications and Postoperative Follow-up after Lung Transplantation in China (2019 edition)* [25]. These documents establish unified technical standards and decision-making frameworks.

Multidisciplinary team development in LTx in China extends beyond traditional models of interdepartmental collaboration. Through institutional mandates and process redesign, the strengths of multiple specialties are integrated into a comprehensive patient management system. This approach is essential not only for optimizing outcomes for individual patients but also for promoting the adoption of homogeneous, high-standard clinical pathways across transplant centers nationwide, thereby providing a structural foundation for sustained quality improvement.

### 2.4. Pillar Four: Establishing a Localized System of Technical Standards to Promote Homogeneous Clinical Practice

To mitigate quality and safety risks in early clinical practice that arose from reliance on individual experience and variability in procedural approaches, a central focus of the LTx management framework in China has been the translation of mature domestic clinical experience into standardized technical specifications. Under the coordination of the Quality Control Center and authoritative bodies such as the Branch of Organ Transplantation of the Chinese Medical Association, expert consensus has been consolidated to systematically develop a comprehensive set of technical standards for LTx. This framework spans the entire clinical continuum and is tailored to the national context and professional characteristics of practice in China. Between 2019 and 2025, a series of influential expert consensuses, clinical guidelines, and technical specifications were issued, for the first time clearly deconstructing and standardizing the LTx process into five core stages. These documents drew on established international guidance, including consensus statements from the ISHLT and the American Thoracic Society (ATS), while also incorporating adaptations relevant to the donor pool, recipient characteristics, and institutional capacities of Chinese transplant centers [7].

First, recipient evaluation and selection: The *Technical Specification for the Selection of Recipient and Preoperative Evaluation of Lung Transplantation in China (2019 edition)* [26] established standardized inclusion and exclusion criteria for transplant candidates.

Second, donor lung procurement and preservation: Documents including the *Technical Specification for Donor Lung Procurement and Protection of Lung Transplantation in China (2019 edition)* [11] and the *Guideline on the Standard of Lung Transplantation Donors and the Acquisition and Transshipment in China* [10] standardized processes for donor evaluation, surgical procurement, quality maintenance, and long-distance transport.

Third, transplant surgical procedures: Standards such as the *Technical Specification for the Operation of Lung Transplantation in China (2019 edition)* [27], the *Guideline on the Application of Extracorporeal Membrane Oxygenation During the Perioperative Period of Lung Transplantation (2019 edition)* [28], and the *Technical Specification for Anesthetic Management of Lung Transplantation in China (2019 edition)* [29] provided detailed guidance on surgical techniques and intraoperative support.

Fourth, perioperative management: Documents including the *Diagnosis and Treatment Specification for Immunosuppressive Therapy and Rejection of Lung Transplantation in China (2019 edition)* [24], the *Clinical Diagnosis and Treatment of Invasive Fungal Mycosis in Chinese Lung Transplant Recipients* [30], and the *Chinese Guideline for Clinical Diagnosis and Treatment of Airway Complications in Lung Transplant Recipients (2024 edition)* [31] established management frameworks for major postoperative complications.

Fifth, long-term follow-up and management of postoperative complications: Guided by the *Technical Specification for Diagnosis and Treatment of Complications and Postoperative Follow-up after Lung Transplantation in China (2019 edition)* [25], a unified model for post-discharge monitoring and intervention was established.

Together, these five interconnected sets of standards constitute a coherent, closed-loop clinical pathway encompassing preoperative, intraoperative, and postoperative care. The establishment and implementation of this technical system mark a transition in LTx in China from an expert-centered, experience-based model to a guideline-oriented, evidence-based approach. By providing transplant centers across diverse regions with clear guidance for key clinical practices, this framework has substantially reduced practice heterogeneity and quality variation attributable to differences among teams or individual clinicians. It has provided a robust technical foundation for the safe and effective dissemination of LTx at scale and represents a central mechanism through which the management system has enabled controlled expansion with sustained quality assurance.

### 2.5. Pillar Five: Establishing Quality-Oriented Evaluation and Dynamic Exit Mechanisms to Drive Continuous Improvement

To ensure the vitality and adaptability of the management system and to systematically guide and incentivize transplant centers to continuously improve quality of care and patient safety, China has established a clear and transparent national framework for quality evaluation and dynamic exit. Grounded in regulatory instruments such as the *Measures for the Administration of Registration of Medical Subjects for Human Organ Transplantation* [32], this framework applies rigorous external evaluation and structured use of assessment results to impose effective constraints and provide positive guidance for institutional behavior. Implementation of this framework is reflected in the following components.

First, institutionalized periodic assessment: Provincial health administrative authorities conduct regular evaluations of transplant centers within their jurisdictions. Assessment domains include compliance with laws and regulations, adherence to management standards, technical capacity, and key indicators of quality and safety. Evaluation of quality and safety relies heavily on the Data Center, with quantitative assessment of the completeness, timeliness, and accuracy of data reporting, as well as close monitoring of postoperative survival, incidence of major complications, and other core outcome and process indicators.

Second, clearly defined exit mechanisms and enforcement procedures: Assessment results are directly linked to institutional authorization to practice and are not merely procedural. For centers that fail to meet evaluation standards, a stepwise set of corrective actions is applied, including mandated rectification within a specified period, suspension of clinical activity, and revocation of authorization. In accordance with regulatory requirements, mandatory exit procedures are initiated for centers that repeatedly fail assessments, report patient survival rates substantially below the national average, or commit serious violations of ethical or technical standards, resulting in formal cancelation of LTx practice credentials.

This dynamic management approach, which allows for both entry and exit, constitutes a critical constraint within the governance framework. By institutionalizing continuous performance pressure across transplant centers, it reinforces patient safety and medical quality as central operational priorities and effectively prevents post-authorization complacency and technical stagnation. Together with the Quality Control Center, Data Center, and physician training bases, this mechanism forms an integrated governance loop encompassing standards guidance, data monitoring, assessment feedback, and dynamic adjustment. It serves as a key safeguard for achieving steady improvements in quality alongside the expansion of LTx activity in China.

### 2.6. Integration of the Five Pillars: A Coherent Management Ecosystem

The five pillars function as interconnected components of a unified management framework rather than as independent structures. The Quality Control Center and Data Center (Pillar One) provide the data infrastructure required to monitor clinical performance, identify areas for improvement, and guide targeted interventions. The National Training System (Pillar Two) translates these identified needs into structured capacity building by supporting the development of qualified professionals and emerging transplant centers. Institutionalized multidisciplinary teams (Pillar Three) integrate this expertise into clinical practice through defined organizational models, whereas standardized technical pathways (Pillar Four) promote consistency in the implementation of clinical protocols across centers. Dynamic evaluation and exit mechanisms (Pillar Five) link registry-based outcome assessment with accreditation and quality oversight, thereby strengthening accountability within the system.

Together, these components form a continuous process of measurement, improvement, and refinement. Improvements in data quality support the development of clinical standards; standardized approaches facilitate training and team development; and enhanced clinical capacity contributes to more consistent patient care. Outcomes generated through this process provide further evidence to refine standards and management strategies. This coordinated framework has accompanied the expansion of lung transplantation capacity in China over the past decade without compromising quality.

## 3. Discussion and Implications: A Management Perspective

The development of LTx in China over the past decade illustrates how coordinated policy design and system-level management can support the expansion of a highly complex medical service while maintaining quality and safety. The experience provides insights not only for transplantation but also for the governance and dissemination of other advanced medical technologies. Unlike mature transplant systems such as those in the United States, where recent reforms have largely focused on refining established allocation and quality-assurance frameworks [5,6], China simultaneously developed its organ donation system, allocation platform, national registry, quality-management infrastructure, and clinical capacity. During the same period, data from the ISHLT registry showed progressive improvements in outcomes and changes in recipient characteristics within largely established institutional structures [7]. The concurrent development of regulatory, operational, and clinical systems distinguishes the Chinese experience and offers a perspective on managing rapid expansion in a technically demanding field.

An additional aspect of this experience is the ethical and regulatory framework supporting organ donation. Reforms implemented after 2015 established a centralized legal and ethical structure administered by the National Health Commission and operationalized through mandatory COTRS-based allocation, with the stated objectives of fairness, transparency, and traceability [1.2]. Independent analyses, including detailed evaluations by Nashan and colleagues, have described the evolution of this framework [2], and recent international reviews have examined its regulatory and clinical development [3]. As in all transplant systems, continued transparency, accessible registry data, and independent oversight remain important for maintaining confidence in outcome reporting and quality governance. Strengthening these elements will likely contribute to the long-term credibility and evaluability of the system.

### 3.1. Alignment with the WHO Framework on Organ Donation and Transplantation

The World Health Assembly Resolution WHA75.9, adopted in 2022, outlines principles for ethical, equitable, and safe transplantation systems. Several features of the Chinese LTx system are consistent with these principles. Key areas of alignment include: (1) Ethical foundation and voluntary donation: The 2015 policy establishing voluntary donation after citizen death as the sole legal source of transplantable organs aligns with WHO principles regarding informed consent and the prohibition of organ trafficking. The transition to a citizen-based donation system and the establishment of a national donation framework reflected an effort to strengthen ethical procurement practices. (2) Equitable allocation: The China Organ Transplant Response System (COTRS) uses mandatory computerized allocation based primarily on medical urgency, geographic considerations, and waiting time. This approach is consistent with WHO recommendations emphasizing equitable access to transplantation. (3) Quality and safety standards: Mandatory accreditation, structured training, and outcome monitoring are supported by national technical specifications covering donor evaluation, procurement, surgical practice, perioperative care, and long-term follow-up. These measures are broadly aligned with WHO recommendations for standardized, evidence-based clinical pathways. (4) Transparency and data-informed governance: The China Lung Transplantation Registry and periodic quality assessments provide mechanisms for performance monitoring and regulatory oversight. Routine reporting, center-level feedback, and accreditation review processes contribute to accountability. (5) Professional development and capacity building: The national training framework, including accredited training centers, standardized curricula, and mentorship programs, is consistent with WHO recommendations regarding workforce competency and transplant-system development.

### 3.2. System-Level Coordination as a Foundation for Quality-Assured Expansion

In fields characterized by technical complexity, ethical sensitivity, and concentrated clinical risk, coordinated planning can help prevent uncontrolled diffusion and support quality assurance. In China, this coordination was achieved through the five-pillar framework described in Section 2. The governance model itself, however, is not the central determinant of success. In China, these functions are administered through centralized governmental structures. In the United States, comparable coordination is achieved through organizations such as OPTN, SRTR, and ISHLT within a more decentralized framework [5,6]. Despite institutional differences, both approaches share several common features: transparent outcome measurement, enforceable quality standards, structured professional training, and clearly defined accountability mechanisms. These principles appear broadly applicable across healthcare systems, whereas the mechanisms used to implement them depend on local institutional contexts.

The Chinese experience also suggests that centralized coordination may facilitate implementation when donation systems, workforce development, and allocation infrastructure must be established simultaneously. Because this review focuses on management and governance, it relies primarily on policy documents, regulations, and registry data that constitute the foundation of the national LTx program. Although this approach is appropriate for system-level analysis, additional independent international evaluations would further enrich understanding of the program’s performance and governance. Such literature remains relatively limited, partly because the centralized system was established only recently and because international dissemination of outcome research typically requires several years.

### 3.3. A Three-Component Pathway Toward Homogeneous Development

The expansion of LTx across 45 geographically distributed centers was supported by three mutually reinforcing elements: transparent outcome measurement, structured workforce development, and standardized clinical practice. Outcome data enabled benchmarking and identification of performance gaps; accredited training programs supported workforce development; and national clinical standards promoted consistency in care delivery. These components are not unique to China. Outcome registries, structured training programs, and evidence-based clinical guidelines are established features of mature transplant systems worldwide. What distinguishes the Chinese experience is the coordinated implementation of these elements during a period of rapid growth, accompanied by improvements in perioperative survival. The relatively small difference in perioperative survival between high-volume and lower-volume centers (84.2% vs. 82.8%) suggests that standardized training and unified clinical pathways may have contributed to reducing inter-center variability. Similar benefits could potentially be achieved in other healthcare systems adopting comparable approaches, regardless of governance structure.

### 3.4. Accountability Mechanisms and Institutional Performance

An important feature of the Chinese model is the integration of registry-based outcome assessment with accreditation decisions. Centers demonstrating persistently unfavorable outcomes, repeated assessment failures, or serious ethical violations may be subject to corrective actions, suspension, or loss of authorization. In Western transplant systems, including OPTN and SRTR, performance monitoring and public reporting are routine, but accreditation consequences are generally implemented through less direct administrative processes. These approaches reflect different regulatory traditions rather than fundamentally different objectives. Public reporting emphasizes transparency and external accountability, whereas direct administrative oversight may provide more immediate incentives for improvement. For countries developing transplant programs, the key consideration is not the specific enforcement model but the existence of meaningful links between outcome measurement and institutional accountability. The form of those links should be adapted to local regulatory capacity and governance structures.

### 3.5. Transferable and Context-Dependent Features of the Chinese Model

China’s experience offers lessons for countries seeking to expand transplant services, although not all elements are equally transferable. Features with broad applicability include registry-based outcome monitoring, standardized training and certification, multidisciplinary team requirements, and unified clinical guidelines. These measures can be implemented across a wide range of healthcare systems and governance arrangements.

Other features are more dependent on local institutional conditions. Centralized government oversight of donor allocation, accreditation, and resource distribution reflects the structure of China’s healthcare system. Comparable objectives are pursued elsewhere through professional networks, accreditation organizations, regional authorities, or public–private partnerships. Likewise, mandatory participation in quality-improvement programs and training initiatives may be enforced through governmental authority, accreditation requirements, reimbursement policies, or professional regulation. Thus, the underlying principles of equity, quality assurance, and accountability appear broadly transferable, whereas implementation strategies require adaptation to local contexts.

### 3.6. Implications for Developing Countries

Between 2015 and 2024, annual LTx volume in China increased from 118 procedures at 9 centers to 925 procedures at 45 centers, while perioperative survival improved from 78.5% to 85.3%. These observations suggest that management systems, including outcome monitoring, standardized processes, workforce development, and accountability mechanisms, may play an important role in supporting safe expansion of complex clinical services.

For countries establishing or expanding transplant programs, several priorities emerge: development of outcome registries, implementation of standardized training pathways, establishment of multidisciplinary-care requirements, adoption of evidence-based clinical guidelines, and creation of mechanisms linking performance measurement to quality improvement. The specific governance model may vary, but transparent data systems and sustained professional development appear to be central components of successful program growth. The Chinese experience suggests that substantial expansion can occur alongside improvements in short-term outcomes when clinical growth is accompanied by coordinated management and quality-assurance systems.

### 3.7. Limitations

Several limitations should be considered. First, although the national registry captures perioperative and medium-term outcomes as well as major complications, CLAD-free survival is not yet recorded as a discrete variable. Inclusion of this endpoint would facilitate comparison with international benchmarks. Second, the registry currently records waiting-list census rather than validated waiting-list mortality, limiting direct comparison with mature international registries. Third, as with all registry-based analyses, data quality depends on reporting completeness and follow-up practices at participating centers. Finally, this management-focused review relies primarily on governmental, regulatory, and institutional sources. Although these sources are appropriate for describing the development of a national system, complementary independent international evaluations would strengthen the evidence base. Future collaborative studies may provide additional insight into long-term outcomes and governance effectiveness.

### 3.8. Future Research Priorities

Several areas warrant further investigation. First, long-term outcomes, particularly CLAD-free survival and predictors of chronic graft dysfunction, should be incorporated into routine registry analyses. Prospective studies examining immunologic mechanisms and graft tolerance may provide additional insight into long-term graft performance. Second, implementation research is needed to identify which quality-improvement strategies are most effective in lower-volume centers and resource-constrained environments. Third, analyses of equity and access should assess whether expansion has benefited patients across geographic regions, socioeconomic groups, and disease categories to a similar extent. Finally, international collaborative studies comparing long-term outcomes between Chinese and other transplant systems, while accounting for differences in recipient characteristics, donor profiles, and follow-up practices, may help clarify the generalizability of these findings.

## 4. Conclusions

Over the past decade, LTx in China has undergone systematic development, progressing from an initial phase of scale accumulation to sustained improvement in quality. The principal factor underlying this progress has been the establishment of a management framework that integrates national-level strategic planning, homogenized technical training, structured multidisciplinary collaboration, standardized clinical pathways, and rigid mechanisms for quality evaluation and program exit. Through a data-driven, standard-guided, and closed-loop management approach, this framework has ensured quality consistency and maintained controllable safety throughout the process of technology dissemination.

Looking ahead, as the field continues to confront challenges such as donor lung scarcity and the increasing age of transplant recipients, this validated management framework is expected to provide ongoing institutional support for the high-quality and sustainable development of LTx. The Chinese experience underscores that, for complex medical technologies, excellence in management systems is as essential as clinical technical capability, and it offers a reference model for the systematic dissemination of comparable technologies worldwide.

## Data Availability

No new data were created or analyzed in this study. Data sharing is not applicable to this article.

## Supplementary Materials

The following supporting information can be downloaded at: https://www.mdpi.com/article/10.3390/healthcare14142075/s1, Table S1: Summary of China’s Lung Transplantation Management System (2015–2024).
