# Supplementary material for: A Decade of Lung Transplantation in China (2015–2024): Driving Quality Improvement and Scale Expansion Through Management Systems
Source: Healthcare (Basel). 2026 Jul 10;14(14):2075. doi: 10.3390/healthcare14142075 (PMC13410501; doi:10.3390/healthcare14142075)
Supplement: Supplementary file 1 [file healthcare-14-02075-s001.zip › healthcare-4309285-supplementary.pdf]

SUPPLEMENTARY TABLE S1

Summary of China's Lung Transplantation Management System (2015–2024)

Pillar Component	Key Institutions/Authority	Primary Functions	Major Standards/Documents
**Pillar 1: Quality Control & Data Infrastructure**			
**National Quality Control Center**	National Health Commission (NHC); Wuxi People's Hospital; China-Japan Friendship Hospital	Accreditation, evaluation, continuous improvement of LTx services	Regulations on the Clinical Application Management of Human Organ Transplantation Technology (2020 edition)<br>Regulations on the Clinical Application Management of Human Organ Transplantation Technology
**National Data Center**	NHC; Wuxi People's Hospital	Standardize data definitions, develop CLuTR, provide feedback reports	Measures for the Management of Human Organ Donation and Transplantation Data
**China Lung Transplant Registry (CLuTR)**	National Data Center	Real-time collection of transplant data, monitoring of quality indicators, outcome tracking	Registry includes: cold ischemia time, complications, perioperative/1-3-5 year survival, infection rates, rejection rates, etc
**Pillar 2: Standardized Training System**			
**Wuxi People's Hospital Training Base**	NHC; Jiangsu Provincial Health Commission; Wuxi People's Hospital	Train LTx surgeons and multidisciplinary professionals; 1-year immersive program; expert-led mentorship	Measures for the Management of Training and Certification of Human Organ Transplant Physicians<br>Basic Requirements for Human Organ Transplant Physician Training Bases (Trial)<br>Training covers: donor evaluation, organ procurement, surgical technique, perioperative management, long-term follow-up
**China–Japan Friendship Hospital Training Base**	NHC; Beijing Municipal Health Commission; China-Japan Friendship Hospital	Train LTx surgeons and multidisciplinary professionals	Same comprehensive curriculum as Wuxi base
**The Second Affiliated Hospital of Zhejiang University School of Medicine Training Base**	NHC; Zhejiang Provincial Health Commission; The Second Affiliated Hospital of Zhejiang University School of Medicine	Train LTx surgeons and multidisciplinary professionals	Same comprehensive curriculum as Wuxi base
**The First Affiliated Hospital of Guangzhou**	NHC; Guangdong Provincial Health Commission; The First Affiliated Hospital of	Train LTx surgeons and multidisciplinary professionals	Same comprehensive curriculum as Wuxi base

**Medical University Training Base**	Guangzhou Medical University		
**Training Outcomes**	Four accredited bases	>230 LTx surgeons and >1,500 multidisciplinary professionals trained and deployed nationwide	Certification of physicians and professionals
**Pillar 3: Multidisciplinary Team Requirements**			
**Mandatory Multidisciplinary Team (MDT) Structure**	NHC	Require all transplant centers to establish formally structured multidisciplinary teams	Regulations on the Clinical Application Management of Human Organ Transplantation Technology (2020 edition)<br>Regulations on the Clinical Application Management of Human Organ Transplantation Technology<br>Core members: thoracic surgery, respiratory/critical care medicine, anesthesiology, intensive care; Extended members: cardiology, rehabilitation, nursing, nutrition, psychology
**Preoperative ICU Optimization**	Individual transplant centers	Early intensive care participation; preoperative clinical optimization	Proportion of recipients admitted to ICU before transplantation: 9.4% (2019) → 18.1% (2024)
**Lifelong Follow-up Led by Respiratory Medicine**	Individual transplant centers; Chinese Medical Association (CMA) guidance	Primary responsibility for long-term care, immunosuppression management, complication diagnosis	Diagnosis and Treatment Specification for Immunosuppressive Therapy and Rejection of Lung Transplantation in China (2019 edition)
**Pillar 4: Standardized Technical Pathways**			
**Recipient Evaluation & Selection**	Branch of Organ Transplantation of CMA	Standardized inclusion/exclusion criteria	Techical Specification for the Selection of Recipient and Preoperative Evaluation of Lung Transplantation in China (2019 edition)
**Donor Lung Procurement & Preservation**	Branch of Organ Transplantation of CMA; National Quality Control Center	Standardized donor evaluation, procurement, quality maintenance, transport procedures	Guideline on the Standard of Lung Transplantation Donors and the Acquisition and Transshipment in China<br>Technical Specification for Donor Lung Procurement and Protection of Lung Transplantation in China (2019 edition)

**Transplant Surgical Procedures**	Branch of Organ Transplantation of CMA; National Quality Control Center	Standardized surgical techniques; intraoperative support protocols	Technical Specification for the Operation of Lung Transplantation in China (2019 edition)<br>Guideline on the Application of Extracorporeal Membrane Oxygenation During the Perioperative Period of Lung Transplantation (2019 edition)<br>Technical Specification for Anesthetic Management of Lung Transplantation in China (2019 edition)
**Perioperative Management**	Branch of Organ Transplantation of CMA	Management of major postoperative complications; immunosuppression; infection prevention	Diagnosis and Treatment Specification for Immunosuppressive Therapy and Rejection of Lung Transplantation in China (2019 edition)<br>Clinical Diagnosis and Treatment of Invasivefungal Mycosis in Chinese Lung Transplant Recipients<br>Chinese Guideline for Clinical Diagnosis and Treatment of Airway Complications in Lung Transplant Recipients (2024 edition)
**Long-term Follow-up & Complication Management**	Branch of Organ Transplantation of CMA	Unified post-discharge monitoring and intervention protocols	Technical Specification for Diagnosis and Treatment of Complications and Postoperative Follow - up after Lung Transplantation in China (2019 edition)
**Pillar 5: Quality Evaluation & Dynamic Exit Mechanisms**			
**Periodic Assessment**	NHC; Provincial Health Commission; National Quality Control Center; National Data Center	Regular evaluation of compliance, adherence to standards, technical capacity, quality/safety indicators	Assessment domains: laws/regulations compliance, standard adherence, technical capacity, perioperative survival, complication rates, data completeness
**Exit Mechanisms**	NHC; Provincial Health Commission; National Quality Control Center; National Data Center	Corrective actions for underperformance: mandated rectification → suspension → revocation of authorization	Mandatory exit initiated for: repeated assessment failure, survival <national average, serious ethical/technical violations
**Data-Driven Feedback Loop**	National Quality Control Center; National Data Center	Individualized quality feedback reports to centers; national-level quality control reports to authorities	Regular statistical analysis and outcome reporting
**China Organ Transplant**	NHC; Big Data Centre of National Health Commission for Human	Computerized, mandatory organ allocation based on	Basic principles and core policies for the allocation and sharing of human organs in China

**Response System (COTRS)**	Tissue, Organ Transplant and Medicine	standardized criteria (medical urgency, waiting time, blood type, geographic distance)	Regulations on the management of procurement and allocation of donated human organs
**Donor Selection Criteria**	National Quality Control Center; National Quality Control Center for Donated Organ Procurement	Standardized criteria for donor lung quality and suitability	Guideline on the Standard of Lung Transplantation Donors and the Acquisition and Transshipment in China<br>Technical Specification for Donor Lung Procurement and Protection of Lung Transplantation in China (2019 edition)
**Accreditation Criteria for New Centers**	NHC	Infrastructure requirements; multidisciplinary team capacity; professional qualifications; compliance with regulations	Measures for the administration of registration of medical subjects for human organ transplantation
**Key Quality Indicators Monitored (CLuTR)**	National Data Center	Cold ischemia time; rates of infection, acute rejection, airway complications; perioperative survival; 1-year, 3-year, 5-year survival; re-transplantation rate; center-specific performance	Monitored annually; used to inform quality feedback and accreditation decisions

Note: CLuTR = China Lung Transplantation Registry; CMA = Chinese Medical Association; COTRS = China Organ Transplant Response System; ECMO = Extracorporeal Membrane Oxygenation; MDT = Multidisciplinary Team; NHC = National Health Commission
